# Predictive characteristics and model development for acute heart failure preceding hip fracture surgery in elderly hypertensive patients: a retrospective machine learning approach

**DOI:** 10.1186/s12877-024-04892-8

**Published:** 2024-03-28

**Authors:** Qili Yu, Mingming Fu, Zhiqian Wang, Zhiyong Hou

**Affiliations:** 1 Department of Geriatric Orthopedics, Third Hospital of Hebei Medical University, Shijiazhuang, 050051 Hebei China; 2 Department of Orthopaedic Surgery, Third Hospital of Hebei Medical University, Shijiazhuang, 050051, 050051 Hebei China; 3 Third Hospital of Hebei Medical University, Shijiazhuang, 050051 Hebei China

**Keywords:** Heart failure, Hypertension, Hip fracture, Preoperative, Nomogram, Prediction model

## Abstract

**Background:**

Hip fractures are a serious health concern among the elderly, particularly in patients with hypertension, where the incidence of acute heart failure preoperatively is high, significantly affecting surgical outcomes and prognosis. This study aims to assess the risk of preoperative acute heart failure in elderly patients with hypertension and hip fractures by constructing a predictive model using machine learning on potential risk factors.

**Methods:**

A retrospective study design was employed, collecting preoperative data from January 2018 to December 2019 of elderly hypertensive patients with hip fractures at the Third Hospital of Hebei Medical University. Using SPSS 24.0 and R software, predictive models were established through LASSO regression and multivariable logistic regression analysis. The models' predictive performance was evaluated using metrics such as the concordance index (C-index), receiver operating characteristic curve (ROC curve), and decision curve analysis (DCA), providing insights into the nomogram's predictive accuracy and clinical utility.

**Results:**

Out of 1038 patients screened, factors such as gender, age, history of stroke, arrhythmias, anemia, and complications were identified as independent risk factors for preoperative acute heart failure in the study population. Notable predictors included Sex (OR 0.463, 95% CI 0.299–0.7184, *P* = 0.001), Age (OR 1.737, 95% CI 1.213–2.488, *P* = 0.003), Stroke (OR 1.627, 95% CI 1.137–2.327, *P* = 0.008), Arrhythmia (OR 2.727, 95% CI 1.490–4.990, *P* = 0.001), Complications (OR 2.733, 95% CI 1.850–4.036, *P* < 0.001), and Anemia (OR 3.258, 95% CI 2.180–4.867, *P* < 0.001). The prediction model of acute heart failure was Logit(P) = -2.091–0.770 × Sex + 0.552 × Age + 0.487 × Stroke + 1.003 × Arrhythmia + 1.005 × Complications + 1.181 × Anemia, and the prediction model nomogram was established. The model's AUC was 0.785 (95% CI, 0.754–0.815), Decision curve analysis (DCA) further validated the nomogram's excellent performance, identifying an optimal cutoff value probability range of 3% to 58% for predicting preoperative acute heart failure in elderly patients with hypertension and hip fractures.

**Conclusion:**

The predictive model developed in this study is highly accurate and serves as a powerful tool for the clinical assessment of the risk of preoperative acute heart failure in elderly hypertensive patients with hip fractures, aiding in the optimization of preoperative risk assessment and patient management.

**Supplementary Information:**

The online version contains supplementary material available at 10.1186/s12877-024-04892-8.

## Introduction

Hip fractures are a significant health concern for the elderly globally, especially among those over the age of 65, where both incidence and mortality rates are relatively high. It is estimated that by 2050, the annual number of hospital admissions due to hip fractures will exceed 4.5 million worldwide as the aging population grows [1]. Elderly women are particularly affected due to osteoporosis, with their risk of hip fractures being two to three times that of elderly men [2]. Epidemiological studies indicate that the occurrence of hip fractures is associated with multiple factors, including age, sex, bone density, history of falls, lifestyle, and chronic diseases. Additionally, the elderly often suffer from various chronic conditions, such as hypertension, diabetes, and heart disease, which not only increase the risk of hip fractures but can also exacerbate post-fracture complications, affecting treatment outcomes and prognosis [3].

Hypertension, a common chronic disease with a relatively high prevalence in the elderly, has a significant impact on the surgical risks and prognosis for patients with hip fractures. Research suggests that patients with hypertension have reduced vascular elasticity and altered hemodynamics, which may increase their risk of acute heart failure preoperatively in the context of hip fractures [4]. Moreover, hypertension is closely associated with various cardiovascular diseases, such as coronary artery disease, myocardial infarction, and valvular heart disease, all of which could further increase the risk of heart failure [5].

Acute heart failure, a severe clinical condition characterized by a rapid decline in the heart's pumping function leading to insufficient perfusion of tissues and organs, can trigger life-threatening complications [6]. Therefore, for elderly patients undergoing hip fracture surgery, the presence of preoperative acute heart failure can directly affect their surgical candidacy, increase surgical risks and postoperative complications, prolong hospital stays, and even raise the mortality rate. Studies show that hip fracture patients with preoperative acute heart failure have a significantly increased mortality rate within one year [7]. Hence, risk assessment for preoperative acute heart failure in elderly patients with hypertension and hip fractures is crucial. Early identification and intervention to optimize the patient's hypertensive and cardiac status can significantly reduce the risk of preoperative acute heart failure, improve surgical outcomes, and enhance patient survival and quality of life. While, we observed that the measurement of heart failure biomarkers like BNP or NT-proBNP is often overlooked by surgeons due to not being included in routine preoperative laboratory tests. This oversight may result in missing patients who have already developed acute heart failure, failing to address this potential high-risk state timely. Despite the limited focus on this area in existing research, the predictive model based on machine learning introduced in this paper holds significant promise. Through in-depth analysis of historical data from our hospital's inpatients and combining clinical and laboratory indicators, we have developed a model to predict the risk of preoperative acute heart failure in elderly patients with hypertension and hip fractures. This model provides robust support for clinical decision-making, enabling personalized medicine and precise treatment.

## Materials and methods

### Study population

We selected medical data from elderly patients who were hospitalized and underwent surgery in our hospital’s orthopedic department between January 2018 and December 2019 through the electronic medical record system. Patients included in this study were aged 65 and above with concurrent hypertension and hip fractures. All patients selected for this research were required to have complete medical records, laboratory test results, and other relevant examination data. Those lacking these documents or not meeting the criteria for hypertension and hip fractures were excluded from the study.

It is noteworthy that this investigation is grounded in a retrospective analysis of existing case data. Rigorous measures were taken to preserve patient confidentiality, including the anonymization of their data, ensuring no breach of privacy. Informed consent was obtained from all subjects and/or their legal guardian(s). This research aligns with the Declaration of Helsinki and has received endorsement and backing from the Institutional Review Board of Hebei Medical University's Third Hospital.

### Definition of disease

Acute heart failure (AHF) is a pathological state characterized by a rapid decline in the heart's pumping capability, leading to insufficient circulatory blood volume to meet the metabolic demands of the body. The European Society of Cardiology defines this condition as an acute change in heart structure or function, accompanied by a significant reduction in cardiac filling and/or ejection efficiency [8]. These changes usually result in the patient experiencing rapid onset of shortness of breath, pulmonary congestion, and systemic organ hypoperfusion. Clinically, a significant increase in the serum levels of BNP or NT-proBNP is one of the key biochemical markers for assessing the diagnosis of acute heart failure [9]. Laboratory Tests: For the diagnosis of acute heart failure, the diagnostic threshold for BNP is set at ≥ 300 pg/mL, applicable across all age groups. For NT-proBNP, we adopt stratified standards to accommodate patients of different age groups: for those under 55 years old, NT-proBNP > 450 pg/mL; for ages 55 to 75 years, NT-proBNP > 900 pg/mL; and for those over 75 years old, NT-proBNP > 1800 pg/mL. The significant elevation of these biochemical markers aids in diagnosing acute heart failure. In distinguishing between an acute episode of heart failure and pre-existing heart failure, we focus on the patient's clinical presentation and the acute changes in biochemical markers (BNP or NT-proBNP) [8].

Hypertension is defined as blood pressure readings that consistently exceed recommended normal levels when measured at rest, without the administration of antihypertensive treatment. According to the latest guidelines by the American Heart Association and the American Society of Hypertension, this condition is specifically characterized by a systolic blood pressure (SBP) reaching or exceeding 130 mmHg, or a diastolic blood pressure (DBP) reaching or exceeding 80 mmHg [10]. Hypertension is not only a common clinical presentation but also an independent risk factor for major health problems such as cardiovascular diseases, cerebrovascular events, and renal dysfunction [10]. Therefore, maintaining blood pressure levels within the normal range is a crucial health management measure to avoid these potential complications.

Definition of Complications: In our study, "complications" are defined as new medical issues that arise from the time of hospital admission to before surgery, excluding acute heart failure. These complications encompass a range of conditions, including cardiovascular complications (such as the risk of myocardial infarction), gastrointestinal complications (like gastric ulcers and gastrointestinal bleeding), endocrine disorders (notably blood sugar fluctuations triggered by stress responses), neurological complications (various cerebrovascular events due to prolonged bed rest and hypertension), and urinary system complications (acute or chronic kidney damage).

### Statistical methods

In this study, to reveal the relationship between acute heart failure and risk factors in elderly patients with hypertension and hip fractures, we initially utilized descriptive statistics to analyze the baseline information of the participants. The normality of continuous variables was confirmed by the Kolmogorov–Smirnov test. Variables with normal distribution were described using means ± standard deviation, whereas non-normally distributed data were presented using medians and interquartile ranges. The distribution characteristics of categorical variables were expressed in frequencies and percentages. The Least Absolute Shrinkage and Selection Operator (LASSO) method was employed to identify risk factors significantly associated with acute heart failure. Based on this, multivariable logistic regression was further utilized to assess these factors' relationships with acute heart failure, leading to the construction of a nomogram for intuitive risk prediction modeling. To evaluate the assumption of collinearity, Variance Inflation Factors(VIF) and tolerances were computed. A VIF value below 5 and a tolerance above 0.1 were used as criteria to signify an absence of notable collinearity. To validate the accuracy and clinical utility of the nomogram model constructed, a series of evaluations were conducted. The model's discriminative ability was measured by the area under the Receiver Operating Characteristic curve (ROC AUC), and its calibration was assessed by comparing predicted outcomes with actual observations using calibration plots. Decision Curve Analysis (DCA) provided standardized net benefits across different risk thresholds to assess the model's practical clinical value, while clinical impact curves depicted the correspondence between identified high-risk patients and actual acute heart failure events at various thresholds. Statistical analysis was performed using SPSS 24.0 and R language. Statistical significance was set at *P* < 0.05.

## Results

### General characteristics of the patients

**Fig. 1:**
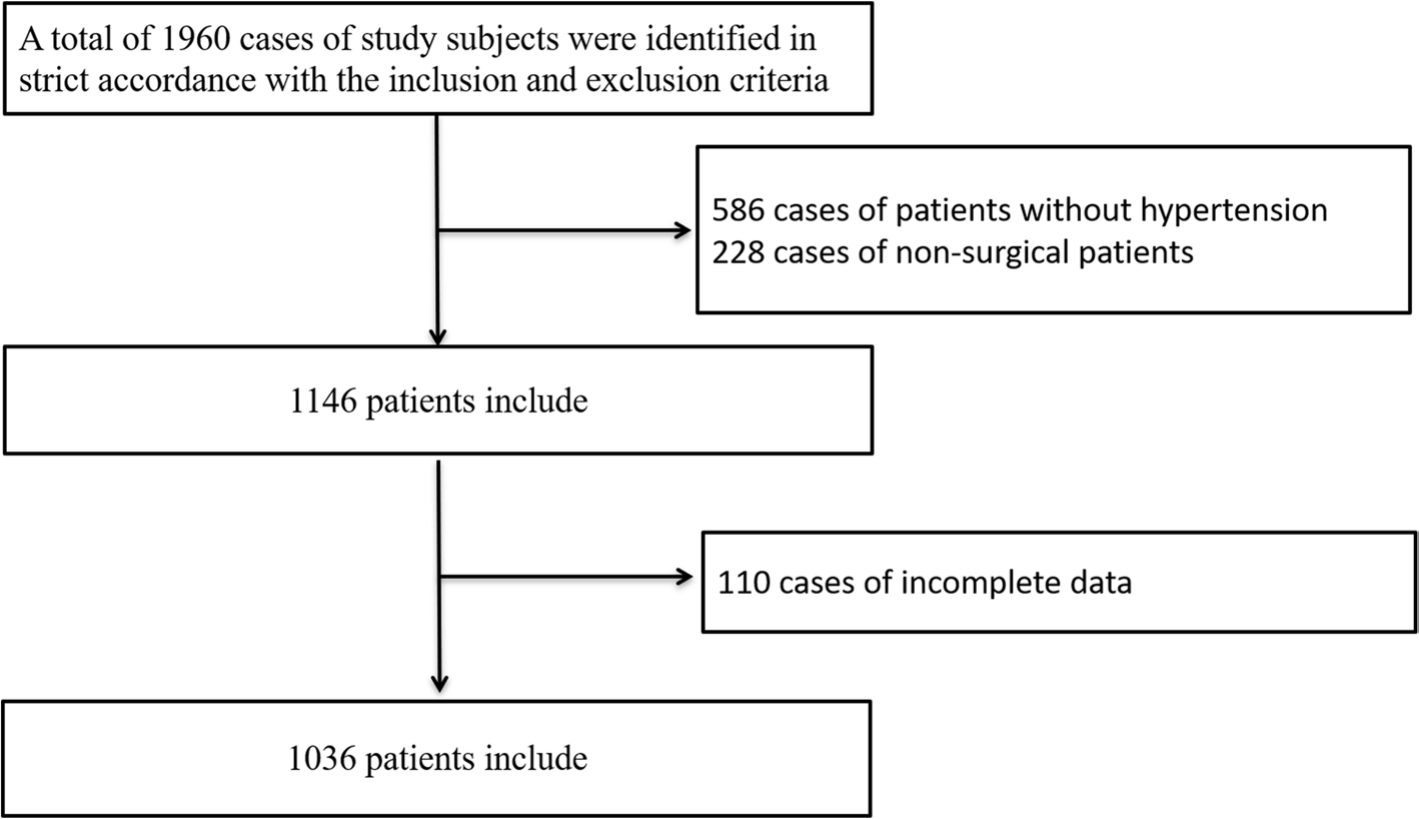
The patient flow chart in our study

Between January 2018 and December 2019, a total of 1960 elderly fracture patients were initially considered for the study. After screening, 922 patients were excluded, resulting in 1038 patients being included in the final analysis. The excluded patients consisted of 586 non-hypertensive individuals, 228 non-surgical patients, and 108 with incomplete data (see Fig. 1). The average age of the 1038 patients was 77.0 ± 7.3 years, with 290 males (27.9%) and 748 females (72.1%). Among the included patients, 190 (18.3%) experienced acute heart failure. The comorbidities primarily included stroke (42.3%), coronary artery disease (32.1%), arrhythmias (6.6%), chronic obstructive pulmonary disease (COPD) (1.6%), cancer (6.4%), and diabetes (30.0%). Notably, 19.4% of all patients developed complications other than acute heart failure during their hospital stay (Table 1). There were statistically significant differences between the heart failure group and the non-heart failure group in terms of gender, age, stroke, arrhythmias, and complications (*P* < 0.05).

**Table 1:** Baseline characteristics of hypertensive patients with hip fracture classified by acute heart failure

Variables	Total (*N* = 1038)	Non-acute heart failure (*N* = 848)	Acute heart failure (*N* = 190)	*P*-value
**Gender, N (%)**				
**Male**	**290(27.9%)**	**256(30.2%)**	**34(17.9%)**	**0.001**
**Female**	**748(72.1%)**	**592(69.8%)**	**156(82.1%)**	
**Age, mean ± SD (years)**	**77.0 ± 7.3**	**76.5 ± 7.1**	**79.6 ± 7.3**	** < 0.001**
**Age group, N (%)**				
< **80 years**	**628(60.5%)**	**546(64.4%)**	**82(43.2%)**	** < 0.001**
≥ **80 years**	**410(39.5%)**	**302(35.6%)**	**108(56.8%)**	
**Comorbidity N (%)**				
**Stroke**				
**Yes**	**439(42.3%)**	**336(39.6%)**	**103(54.2%)**	** < 0.001**
**No**	**599(57.7%)**	**512(60.4%)**	**87(45.8%)**	
**Coronary heart disease**				
**Yes**	**333(32.1%)**	**261(30.8%)**	**72(37.9%)**	**0.058**
**No**	**705(67.9%)**	**587(69.2%)**	**118(62.1%)**	
**Diabetes**				
**Yes**	**311(30.0%)**	**261(30.8%)**	**50(26.3%)**	**0.225**
**No**	**727(70.0%)**	**587(69.2%)**	**140(73.7%)**	
**COPD**				
**Yes**	**17(1.6%)**	**13(1.5%)**	**4(2.1%)**	**0.574**
**No**	**1021(98.4%)**	**835(98.5%)**	**186(97.9%)**	
**Cancer**				
**Yes**	**82(6.4%)**	**53(5.6%)**	**29(8.9%)**	**0.969**
**No**	**1191(93.6%)**	**893(94.4%)**	**298(91.1%)**	
**Arrhythmia**				
**Yes**	**69(6.6%)**	**46(5.4%)**	**23(12.1%)**	**0.001**
**No**	**969(93.4%)**	**802(94.6%)**	**167(87.9%)**	
**Complications**				
**Yes**	**201(19.4%)**	**125(14.7%)**	**76(40.0%)**	** < 0.001**
**No**	**837(80.6%)**	**723(85.3%)**	**114(60.0%)**	

Values are presented as mean ± standard deviation, median (interquartile range), or number (percentage) as appropriate, *SD* Standard deviation, *COPD* Chronic Obstructive Pulmonary Disease

### Univariate analysis of laboratory data and ultrasound examination

In analyzing laboratory and lower limb venous ultrasound examination results of elderly patients with hypertension and hip fractures, it was found that cases with acute heart failure had significantly higher proportions of anemia, hypokalemia, hyponatremia, and hypoalbuminemia compared to those without acute heart failure. Additionally, the incidence of lower limb venous thrombosis was significantly higher in the acute heart failure group than in the non-heart failure group (Table 2). These differences were statistically significant (*P* < 0.05).

**Table 2:** The results of univariate analysis of laboratory data and ultrasound examination

Variables	Total (*N* = 1038)	Non-acute heart failure (*N* = 848)	Acute heart failure (*N* = 190)	*P*-value
Anemia				
Yes	**328(31.6%)**	**210(24.8%)**	**118(62.1%)**	** < 0.001**
No	**710(68.4%)**	**638(75.2%)**	**72(37.9%)**	
Hypokalemia				
Yes	**154(14.8%)**	**107(12.6%)**	**47(24.7%)**	** < 0.001**
No	**884(85.2%)**	**741(87.4%)**	**143(75.3%)**	
Hyponatremia				
Yes	**213(20.5%)**	**148(17.5%)**	**65(34.2%)**	** < 0.001**
No	**825(79.5%)**	**700(82.5%)**	**125(65.8%)**	
Hypoalbuminemia				
Yes	**350(33.7%)**	**244(28.8%)**	**106(55.8%)**	** < 0.001**
No	**688(66.3%)**	**604(71.2%)**	**84(44.2%)**	
Lower extremity venous thrombosis				
Yes	**359(34.6%)**	**279(32.9%)**	**80(42.1%)**	**0.016**
No	**679(65.4%)**	**569(67.1%)**	**110(57.9%)**	

Values are presented as median (interquartile range), or number (percentage) as appropriate

### Construction of a predictive model based on risk factors

**Fig. 2:**
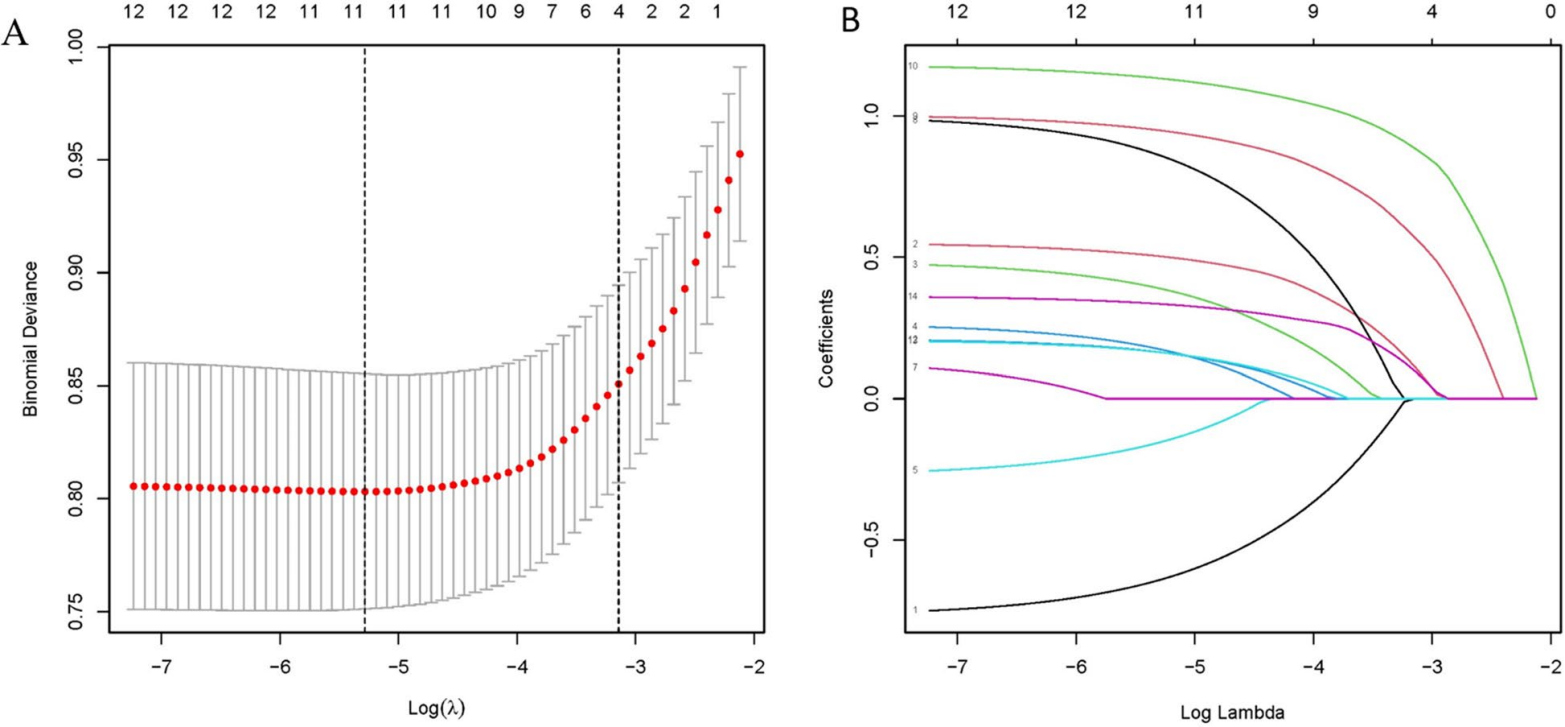
Data statistics and clinical feature selection using the LASSO binary logistic regression model. **A** Optimal parameter (lambda) selection in the LASSO model used fivefold cross-validation via minimum criteria. The partial likelihood deviance (binomial deviance) curve was plotted versus log(lambda). **B** LASSO coefficient profiles of the 14 features. A coefficient profile plot was produced against the log(lambda) sequence

**Fig. 3:**
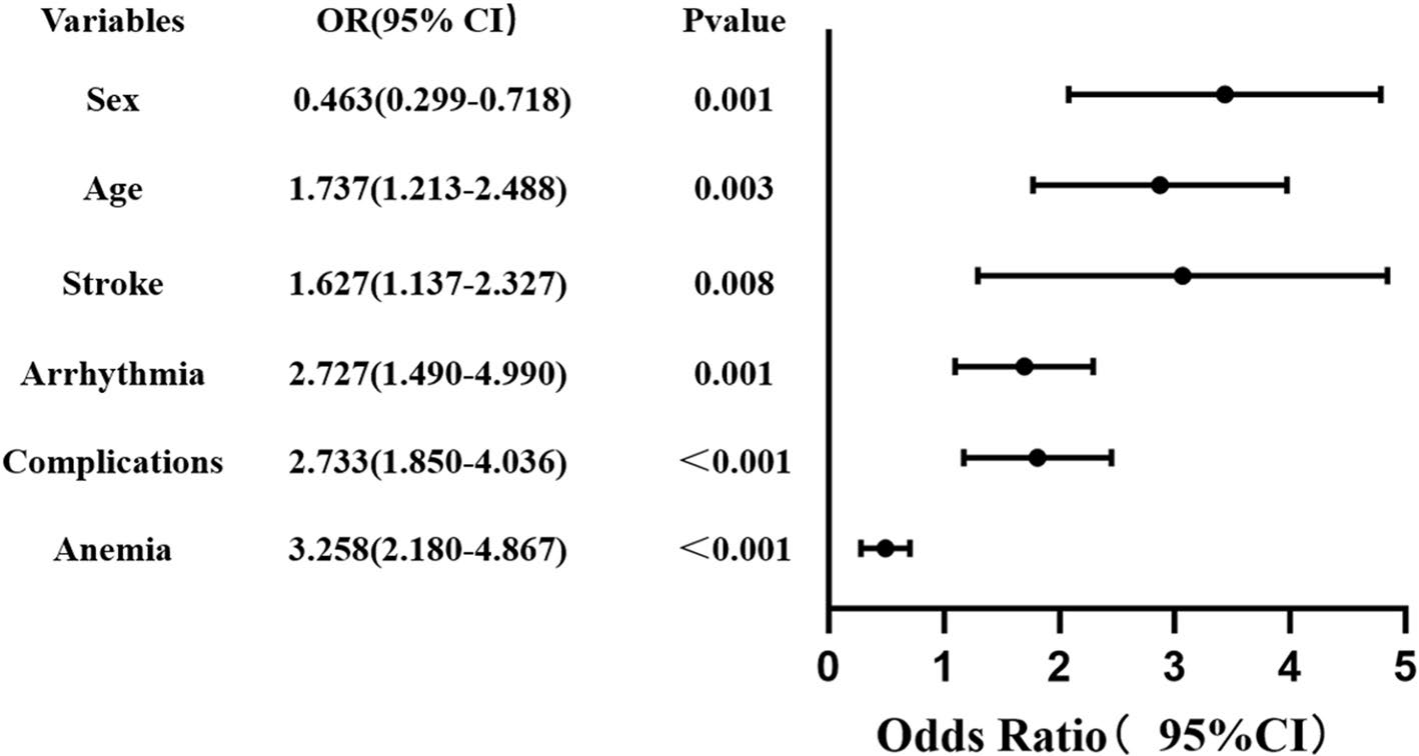
Forest plot showing the relationship between risk factors and the occurrence of preoperative acute heart failure in elderly patients with hypertension combined with hip fracture

**Fig. 4:**
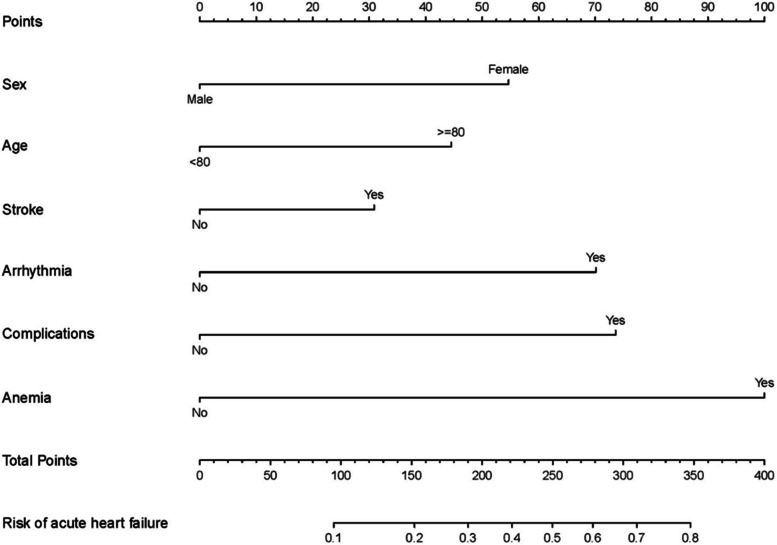
A nomogram model for predicting the occurrence of preoperative acute heart failure in elderly patients with hypertension combined with hip fractures

Through the LASSO regression model, 14 variables were selected from the cohort, and 11 of these were chosen as predictors in the model (Fig. 2A and B). Multivariable logistic regression analysis revealed that gender, age, stroke, arrhythmias, anemia, and complications are independent risk factors for preoperative acute heart failure in elderly patients with hypertension and hip fractures (Table 3 and Fig. 3). Based on these risk factors, we constructed a nomogram model to predict the probability of preoperative acute heart failure in elderly patients with hypertension and hip fractures (see Fig. 4). The prediction model of acute heart failure was Logit(P) = -2.091–0.770 × Sex + 0.552 × Age + 0.487 × Stroke + 1.003 × Arrhythmia + 1.005 × Complications + 1.181 × Anemia. We have calculated the Variance Inflation Factor (VIF) within our model, and the results show that all predictor variables have VIF values well below the commonly used threshold of 5, specifically: Gender (Sex) 1.01, Age 1.02, Stroke 1.02, Arrhythmia 1.01, Complications 1.04, and Anemia 1.01.

**Table 3:** Prediction factors of preoperative acute heart failure in geriatric patients with hip fracture

	B	SE	Wald	*P* value	Odds ratio	95%CI
Sex	-0.770	0.224	11.815	0.001	0.463	0.299–0.718
Age	0.552	0.183	9.071	0.003	1.737	1.213–2.488
Stroke	0.487	0.183	7.099	0.008	1.627	1.137–2.327
Arrhythmia	1.003	0.308	10.589	0.001	2.727	1.490–4.990
Complications	1.005	0.199	25.509	< 0.001	2.733	1.850–4.036
Anemia	1.181	0.205	33.325	< 0.001	3.258	2.180–4.867
Constant	-2.091	0.215	182.383	< 0.001	0.055

### Validation and assessment of the nomogram

**Fig. 5:**
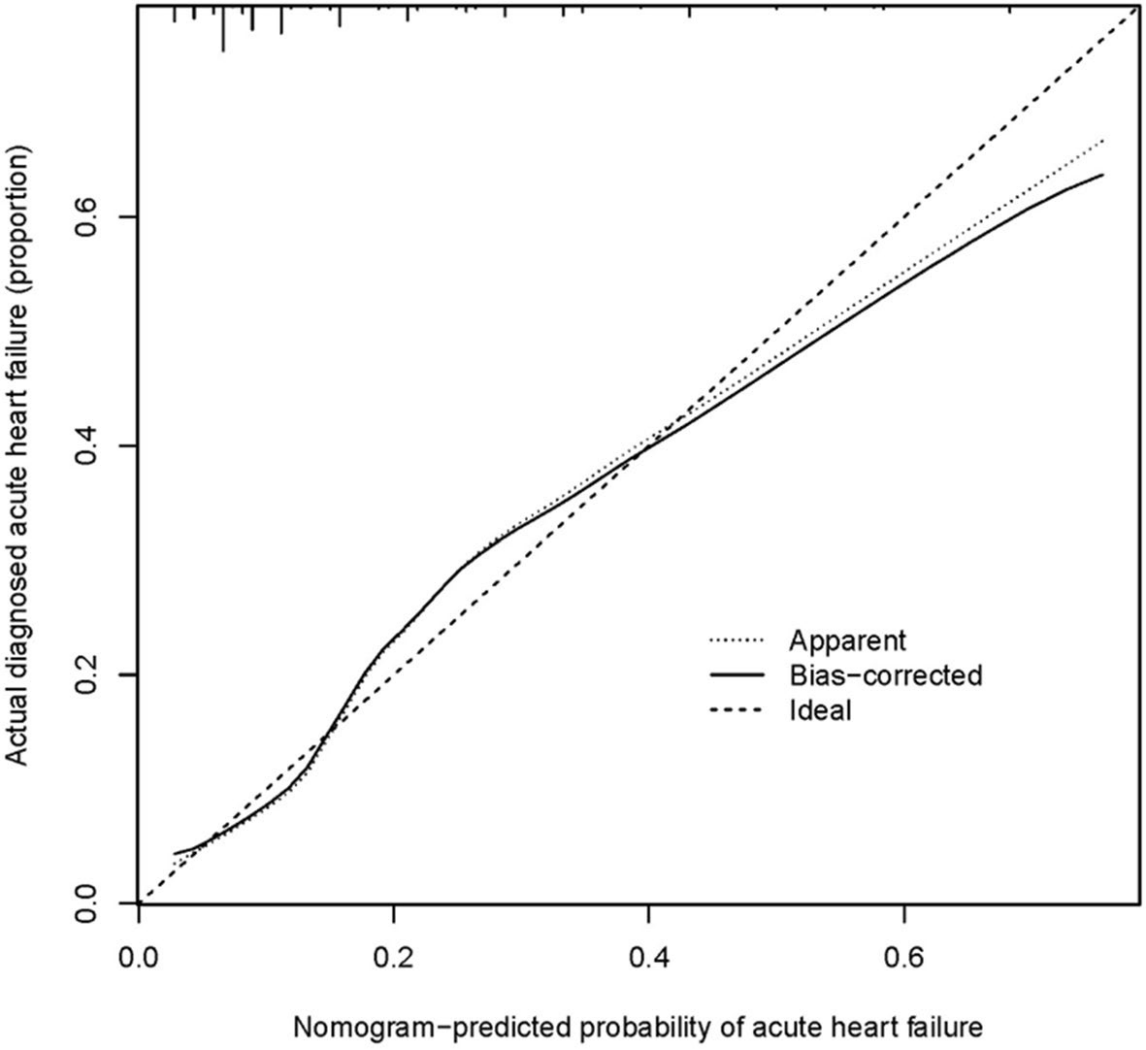
Calibration curves of the acute heart failure nomogram prediction in the cohort. The x-axis represents the predicted acute heart failure risk. The y-axis represents the actual diagnosed acute heart failure. The diagonal dotted line represents a perfect prediction by an ideal model. The solid line represents the performance of the nomogram, of which a closer fit to the diagonal dotted line represents a better prediction

**Fig. 6:**
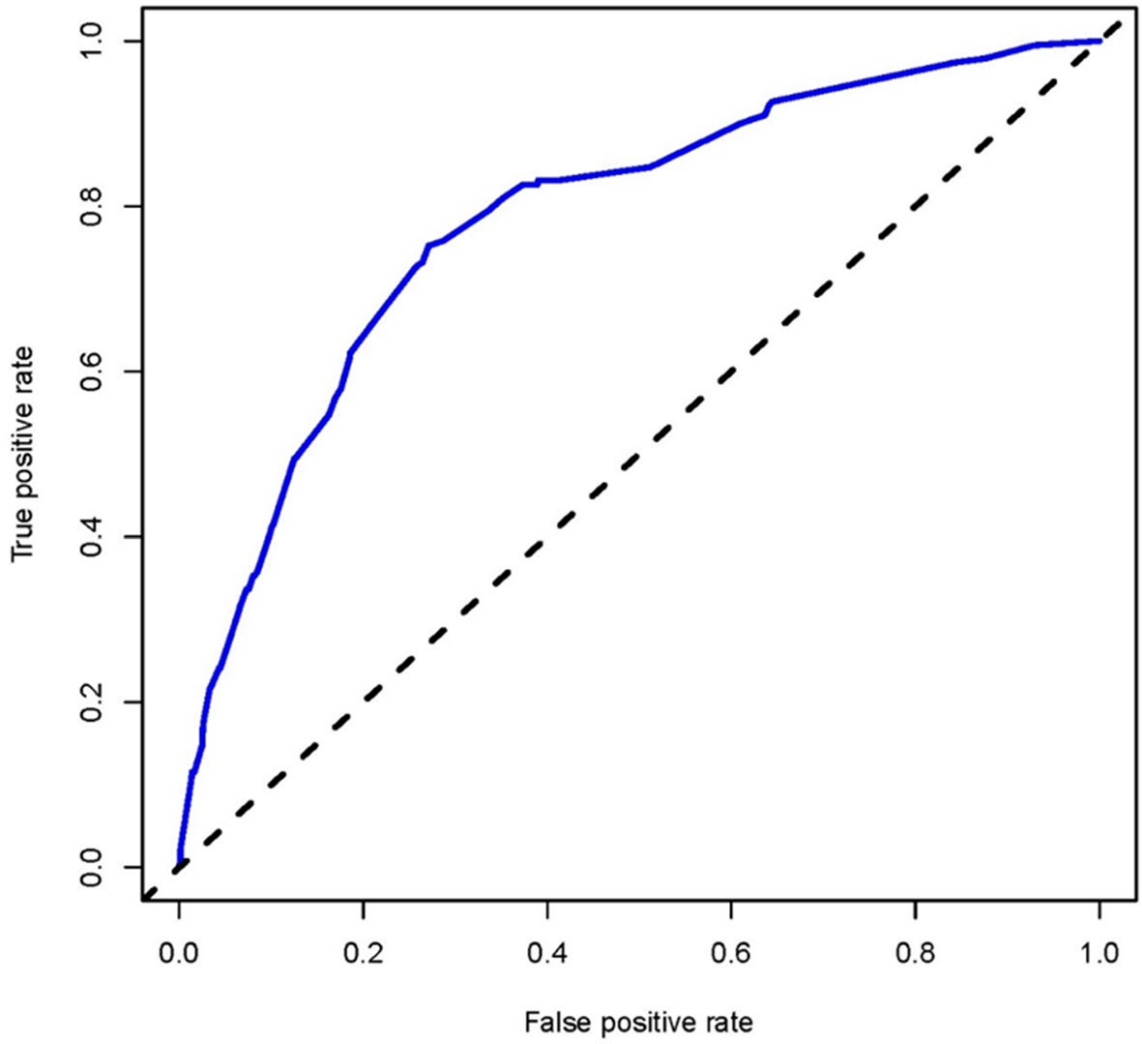
Analysis of ROC curve for the predictive values of preoperative acute heart failure

In this study, the nomogram was validated using the Bootstrap method with 1000 resamples. The validation demonstrated that the calibration curve of the nomogram deviated only slightly from the ideal line, indicating good consistency between the predicted outcomes and the observed results (Fig. 5). To assess the predictive performance of the nomogram, we evaluated it using the Receiver Operating Characteristic (ROC) curve and calculated an Area Under the Curve (AUC) of 0.785 (95% CI, 0.754–0.815) (Fig. 6). Furthermore, the nomogram model's corrected C statistic obtained through Bootstrap resampling was 0.776, indicating good performance in internal validation. This means the model has strong discriminative ability and can accurately predict patients' risk in the acute heart failure risk nomogram.

### Clinical application

**Fig. 7:**
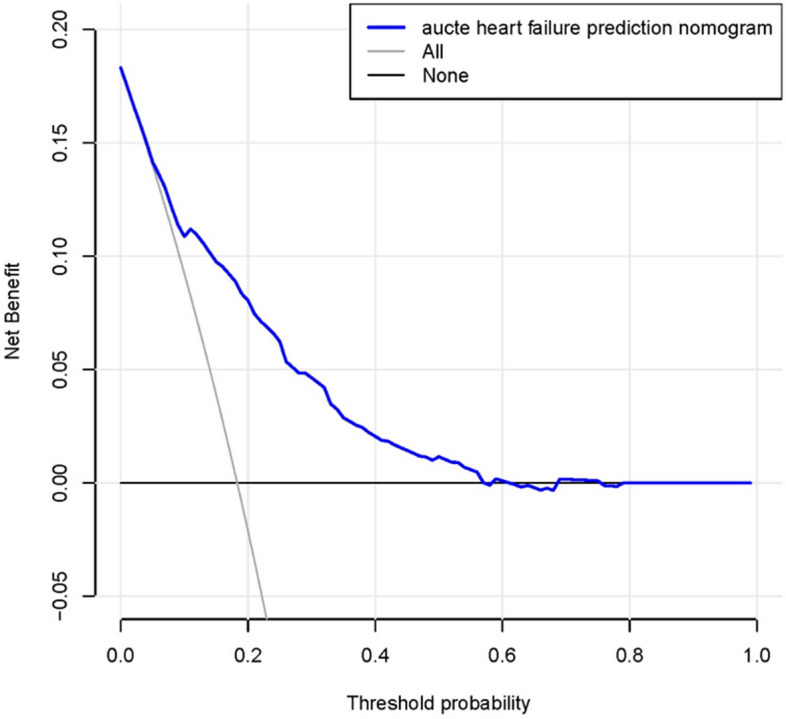
Decision curve analysis for the acute heart failure nomogram. The y-axis measures the net benefit. The blue line represents the acute heart failure risk nomogram. The thin solid line represents the assumption that all patients have acute heart failure, while the thin thick solid line represents the assumption that no patients have acute heart failure. This indicates that using the nomogram to guide clinical intervention can provide a positive net benefit to patients within a cutoff value probability range of 3% to 58%

Figure 7 presents the decision curve analysis results for the acute heart failure nomogram. The decision curve showed that the optimal cutoff value probability range of 3% to 58%. This indicates that the predictive model has high potential clinical utility in forecasting the occurrence of preoperative acute heart failure in elderly patients with hypertension and hip fractures. The model provides robust support for clinicians in making decisions, being able to predict with a certain degree of accuracy whether these patients will experience acute heart failure.

## Discussion

In this study, we conducted a comprehensive analysis of clinical data from 1038 patients with hypertension and hip fractures to construct a model predicting the risk of preoperative acute heart failure. Multivariable logistic regression analysis identified several key independent predictive factors, including patient gender, age, history of stroke, arrhythmias, anemia, and the presence of other complications. Based on these factors, we developed a nomogram tool to facilitate the assessment of patient risk levels by clinicians. To validate the effectiveness of this predictive model, internal validation methods were employed. The validation results indicated that the model has a high degree of calibration, meaning it can accurately predict the occurrence of the event; it also demonstrated excellent discriminative ability, signifying that the model can effectively differentiate between high and low-risk patients. Moreover, the clinical utility of the model was confirmed, allowing clinicians to improve preoperative assessments and management of patients with hypertension and hip fractures.

In our research, it was discovered that among elderly patients with hypertension and heart failure, women were more likely than men to develop acute heart failure. This phenomenon may be linked to physiological changes and variations in hormone levels [11]. Epidemiological studies have shown that after menopause, women experience a significant decrease in estrogen levels, a hormone that protects against vascular inflammation and atherosclerosis. The reduction in estrogen may lead to a decrease in cardiovascular protection, thereby increasing the risk of acute heart failure [11, 12]. Besides hormonal factors, women's cardiac structure and cardiovascular physiology also undergo specific changes with aging. Studies have indicated that compared to men, women experience an earlier decline in ventricular diastolic function and an increase in ventricular wall thickness, which may increase the risk of heart failure on the basis of hypertension [13]. Furthermore, women with heart disease often exhibit unique clinical symptoms, such as being more likely to present as asymptomatic or with atypical symptoms compared to men, potentially leading to delayed diagnosis and exacerbating the severity of heart failure.

It is well-known that with advancing age, the heart undergoes a series of structural and functional changes, presenting significant challenges to its adaptability and compensatory mechanisms [14]. Specifically, physiological changes common in the hearts of elderly patients, such as ventricular wall thickening, reduced left ventricular diastolic function, and increased cardiac filling pressures, are direct contributors to heart failure. These changes have a more pronounced impact on cardiac function under the condition of hypertension. The persistent effect of hypertension significantly increases the cardiac load, leading to ventricular hypertrophy as a means to maintain the ability to counteract the increased vascular resistance. Although this compensatory mechanism is effective in the short term, in the long run, it exacerbates the abnormality in cardiac structure, such as the progression of myocardial fibrosis, a process widely recognized in medical research. Researchers like Kurdi have pointed out that prolonged hypertension can cause disarray in cardiac structure and function, leading to a continuous decline in cardiac pump efficiency [15]. Additionally, systemic arterial stiffening, which is common in elderly patients, increases peripheral resistance during cardiac pumping. This arterial stiffening is closely associated with hypertension, as high blood pressure accelerates the calcification of the vessel wall and the deposition of collagen fibers. Under such circumstances, the heart, in an attempt to overcome the increased vascular resistance, endures an increasing load, potentially leading to functional failure [16]. At the cellular level, the increase in age-associated cardiomyocyte apoptosis and a decline in myocardial cell repair capacity further aggravate cardiac functional decline. Hypertension accelerates this process as it promotes oxidative stress and inflammatory responses, known to impact the survival and function of myocardial cells. While age itself is a significant risk factor for heart failure, the presence of hypertension undoubtedly exacerbates this risk [17]. Therefore, in elderly patients with hypertension, comprehensive assessment and optimization of blood pressure control are especially crucial before implementing hip fracture surgery to minimize the risk of acute heart failure.

Stroke is an important predictor of heart failure in elderly patients with hypertension, with studies showing that a history of stroke can lead to an increased incidence of heart failure. In one study, a history of stroke was identified as one of the independent risk factors for predicting heart failure in patients with cardiac disease [18]. A stroke is typically caused by acute cerebral ischemia due to cerebral vascular occlusion, which not only exposes the patient's systemic arteriosclerosis but may also reflect widespread cardiovascular dysfunction. Hypertension, as a major risk factor for arteriosclerosis, can lead to structural and functional changes in the heart over time, such as left ventricular hypertrophy, ventricular remodeling, and reduced cardiac compliance. These changes increase the workload on the heart and may ultimately lead to heart failure. The neuroendocrine system plays a significant role in the development of both stroke and heart failure. Studies have found that increased sympathetic nervous activity following a stroke leads to a further increase in cardiac load, promoting the development of heart failure [19]. In addition, inflammatory responses are also linked to the association between stroke and heart failure. It has been shown that the inflammatory pathways activated after a stroke may further impact cardiac function by damaging endothelial function and promoting myocardial fibrosis [20]. Therefore, in managing elderly hypertensive patients, it is crucial to understand and assess the impact of stroke history on the risk of heart failure and to consider this factor in treatment strategies. Maintaining blood pressure at optimal levels, reducing the progression of arteriosclerosis, and suppressing sympathetic nervous activity and inflammatory responses with appropriate medication can help lower the risk of these patients developing heart failure. However, further research is needed to select more suitable treatment methods to reduce the occurrence of acute heart failure.

Arrhythmias, especially in elderly patients with hypertension, are a significant risk factor for the exacerbation of heart failure. In hypertensive patients, the heart, due to long-standing high blood pressure, undergoes ventricular hypertrophy and cardiac remodeling. These changes affect the electrophysiological properties of the heart, increasing the risk of arrhythmias [21]. Arrhythmias such as atrial fibrillation are particularly common in elderly patients with hypertension, and these irregularities reduce cardiac pumping efficiency by decreasing ventricular filling time and increasing irregular contractions of the heart. Moreover, in hypertensive patients, changes in cardiac structure and reduced vascular compliance lead to a decrease in cardiac output. Arrhythmias further amplify this effect, particularly at high ventricular rates, where effective cardiac output significantly drops, potentially leading to the rapid onset or worsening of heart failure symptoms [22]. Additionally, arrhythmias themselves can cause myocardial ischemia, which in elderly hypertensive patients with existing cardiac remodeling and coronary artery changes, can exacerbate myocardial damage and further promote the development of heart failure [23]. In clinical practice, the management strategy for elderly hypertensive patients with hip fractures should pay special attention to the monitoring and control of arrhythmias. Aggressive antiarrhythmic treatment, combined with strict blood pressure control, may help to reduce the incidence of heart failure in these patients and improve their quality of life. Therefore, when assessing and treating elderly hypertensive patients with hip fractures, arrhythmias should be considered a potential pathologic pathway mediating heart failure, and appropriate preventative and interventional measures should be taken.

Anemia is exceedingly common in elderly patients with hip fractures, occurring in 31.6% of patients in our study, partly due to bleeding associated with the hip fracture. Previous research has indicated that anemia is an independent risk factor for heart failure in elderly patients with hypertension, likely due to an imbalance in oxygen supply and demand [24]. In the state of anemia, the reduced red blood cell count consequently decreases the blood’s oxygen-carrying capacity, leading to insufficient oxygen supply to tissues and organs, particularly the heart. To compensate for the deficit in oxygen transport, the heart must work harder by increasing heart rate and contractility to boost cardiac output. Functionally, this manifests as an increased cardiac load, which, over time, can lead to myocardial damage and accelerate the development of heart failure [25]. In elderly patients with hypertension, where the heart is already under increased afterload, anemia further raises the heart's oxygen demand while simultaneously reducing effective oxygen delivery, creating a supply–demand imbalance within myocardial cells. This imbalance may promote ventricular hypertrophy, exacerbate systolic dysfunction, and ultimately lead to acute heart failure [26]. Moreover, hypertension is closely associated with chronic kidney disease, which often leads to anemia because the kidneys are the primary site of erythropoietin production. Thus, when elderly hypertensive patients suffer a hip fracture, acute blood loss due to the fracture may increase the risk of acute heart failure. Simultaneously, chronic kidney disease's inability to produce adequate erythropoietin forms a vicious cycle, heightening the risk of acute heart failure [27]. In the clinical treatment of elderly hypertensive patients with hip fractures, anemia should be given sufficient attention, with timely assessment and close monitoring of hemoglobin levels, and measures to correct anemia when necessary may be significant in reducing cardiac burden and delaying the progression of acute heart failure.

In elderly patients with hypertension, the occurrence of complications following a hip fracture significantly increases the risk of acute heart failure. Particularly in the respiratory system, common post-fracture pulmonary complications such as pneumonia and pulmonary embolism can cause hypoxemia and increased cardiac stress, further burdening the already strained heart due to hypertension [28]. Digestive system complications, such as gastric ulcers and gastrointestinal bleeding, may be exacerbated by the prolonged bed rest and use of analgesics necessary after hip fracture, leading to reduced blood volume and altered cardiac preload [29]. Endocrine system disturbances, especially fluctuations in blood sugar triggered by stress responses, can intensify the metabolic pressure on the heart [30]. In terms of neurological complications, the risk of various cerebrovascular events increases with prolonged bed rest and vascular damage caused by hypertension, which can lead to the occurrence of acute heart failure [31]. In the urinary system, acute or chronic renal injury may cause volume overload and the accumulation of metabolic products, exacerbating the likelihood of acute heart failure [32]. Complications in the circulatory system, such as the risk of myocardial infarction, also increase due to traumatic stress and prolonged bed rest, especially in hypertensive patients with reduced vascular elasticity [33]. Therefore, in the context of hypertension, complications in these organ systems in elderly patients with hip fractures can all become significant risk factors for acute heart failure, necessitating close monitoring and prevention.

## Limitations

This study has several limitations that warrant attention. Firstly, it is a non-randomized retrospective analysis, which may lead to potential comparative biases, such as sample selection and inclusion biases. Although we have endeavored to control these biases, we cannot eliminate the possible impacts they may have had. Secondly, since our observation and analysis were limited to existing data, we might not have captured other important variables that could influence the study outcomes. These unconsidered variables may have an effect on the results. Thirdly, this study only analyzed preoperative acute heart failure in elderly patients with hypertension and hip fractures and did not include patients who developed acute heart failure postoperatively. Therefore, further research is needed to investigate these patients to assess the risk factors that might lead to acute heart failure during the entire perioperative period. Despite these limitations, our study still provides valuable insights, and future research could improve upon this by expanding the sample size, considering more potential risk factors, and conducting long-term follow-ups to validate the predictive ability of the model.

## Conclusion

In this study, we developed a predictive model based on logistic regression to forecast the risk of preoperative acute heart failure in elderly patients with hypertension and hip fractures. The model employs indicators such as age, gender, history of stroke, arrhythmias, complications, and anemia as predictive factors. Through individualized analysis of these indicators, we can more accurately assess the risk of acute heart failure before surgery. These indicators are relatively easy to obtain and provide clinicians with a basis for early intervention and the potential to reduce the incidence of acute heart failure.

### Supplementary Information

**Supplementary Material 1.**

**Supplementary Material 2.**

## Data Availability

Data is provided within the supplementary information files.
